# Cathodal tDCS and robotic therapy for upper limb rehabilitation in chronic stroke: a randomized controlled trial

**DOI:** 10.3389/fneur.2025.1644855

**Published:** 2025-11-19

**Authors:** Jin Chen, Jingang Du, Chunfang Wang, Hongli Yu

**Affiliations:** 1 Department of Rehabilitation Medical, The Third Central Hospital of Tianjin, Tianjin, China; 2 Tianjin Union Medical Centre, Tianjin Rehabilitation Institute, Tianjin, China; 3 Tianjin Key Laboratory of Bioelectromagnetic Technology and Intelligent Health, Hebei University of Technology, Tianjin, China

**Keywords:** cathodal transcranial direct current stimulation, robotic therapy, stroke, upper limb rehabilitation, primary motor cortex

## Abstract

**Introduction:**

This randomized controlled trial evaluated the effect of contralesional cathodal transcranial direct current stimulation (ctDCS) combined with robotic therapy (RT) on upper limb recovery in patients with chronic subcortical ischemic stroke.

**Methods:**

Thirty-one participants were randomized to receive either active ctDCS or sham stimulation during RT, administered five times per week for four weeks. Outcomes were assessed using clinical rehabilitation scales and robotic evaluation of movement parameters at baseline, immediately post-intervention, and at two-week follow-up.

**Results:**

The active group demonstrated significantly greater improvement in Upper Extremity Fugl-Meyer Assessment, with a between-group difference of 4.61 (95% CI: 51.36 to 55.46, p = 0.023) post-intervention. Functional efficiency (mean difference: 1.82, 95% CI: 9.13 to 12.00) and movement speed (mean difference: 3.46, 95% CI: 51.60 to 56.74) also favored the active group.

**Conclusion:**

These findings suggest that combining ctDCS with RT may enhance the efficiency of specific upper limb motor tasks in patients with chronic subcortical ischemic stroke, compared to RT alone.

## 1 Introduction

Stroke remains a leading global cause of adult disability, placing a substantial burden on healthcare systems and society. Upper limb dysfunction affects over 80% of stroke survivors, with 30–60% continuing to experience motor impairments 6 months post-stroke. These deficits often limit functional recovery and restrict activities of daily living and social participation (1, 2). Given the limitations of current rehabilitation approaches, developing effective strategies to restore upper limb function represents a critical challenge for clinicians and researchers.

Transcranial direct current stimulation (tDCS) has emerged as a promising non-invasive brain stimulation technique to address the pathophysiological basis of post-stroke motor impairment, particularly the interhemispheric imbalance (3). This model posits that stroke-induced disruption of cortical excitability balance results in excessive inhibition of the damaged hemisphere by the unaffected one. tDCS uses a weak direct current to modulate cortical plasticity and facilitate neural reorganization, offering advantages of operational simplicity and safety (37). While the widely applied anodal tDCS protocol aims to increase excitability of the ipsilesional motor cortex, its therapeutic outcomes have shown considerable variability (4, 5), prompting the search for more optimized protocols. An alternative approach involves applying cathodal tDCS (ctDCS) to the contralesional hemisphere, intending to suppress its hyperexcitability and thereby reduce its inhibitory influence, which may help normalize interhemispheric dynamics and promote recovery (6). However, the effects of tDCS are strongly dependent on stimulation parameters, and the evidence for the efficacy of ctDCS specifically remains less established and requires further rigorous investigation (7, 8).

The combination of neuromodulation techniques like tDCS with targeted motor training is theorized to yield synergistic effects for promoting sustained recovery (9). Robotic therapy (RT) is particularly suited for this integration, as it enables the delivery of high-intensity, repetitive, and task-oriented training—a cornerstone for driving neuroplasticity—with exceptional precision and consistency (10). While the pairing of tDCS and RT is conceptually sound, the specific effects of ctDCS combined with RT have not been sufficiently explored. Therefore, this study is designed to resolve this specific research problem by conducting a single-blind, randomized, controlled trial. Our primary objective is to definitively evaluate whether the combined application of ctDCS and RT leads to superior improvements in upper limb motor function in chronic stroke patients compared to RT alone, thereby providing robust evidence to inform optimal clinical rehabilitation protocols.

## 2 Materials and methods

### 2.1 Participants

This study was approved by the ethics committee of Nankai University (No. NKUIRB2018016) and have been performed in accordance with the Declaration of Helsinki. All methods were performed in accordance with the relevant guidelines and regulations. The participants were comprehensively informed about the experiment, including the possibility of minor adverse effects related to tDCS, such as transient itching, burning, and scalp prickling. All participants or their guardians signed an informed consent form before enrollment.

**Figure 1:**
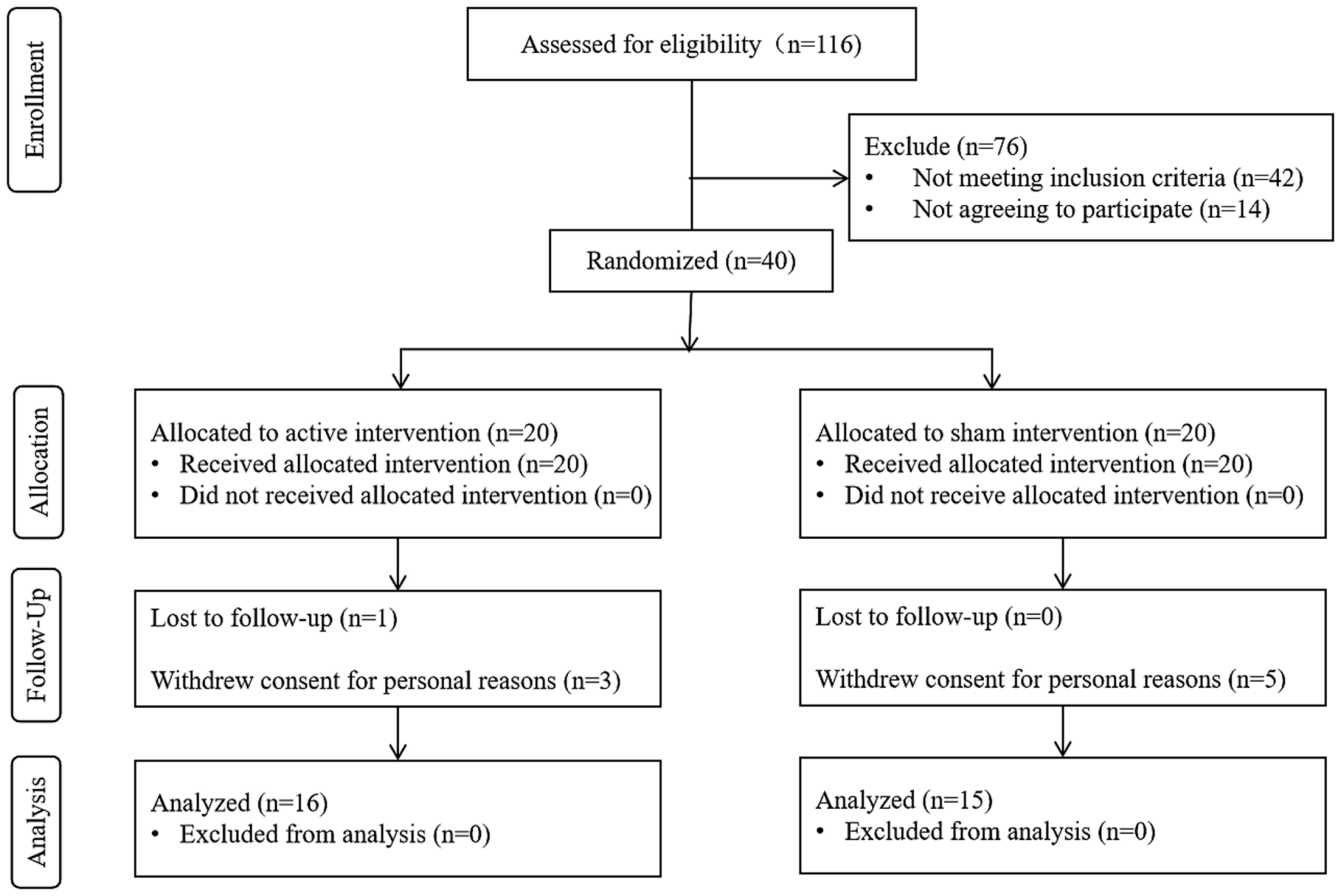
Consort flow diagram of this study.

Thirty-one patients with chronic stroke recruited from Tianjin Union Medical Centre were included. A consort flow diagram is shown in Figure 1. The participants were randomly divided into an active group (*n* = 16) or a sham group (*n* = 15) using a random number table. The random allocation sequence was computer-generated by an independent statistician using MATLAB. A blocked randomization (block size of 4) was used to ensure a balanced group size throughout the recruitment period. The generated allocation sequence was concealed using sequentially numbered, opaque, sealed envelopes (11). The inclusion criteria of the patients were as follows: ① patients aged 50–70 years, ② patients with a first diagnosis of unilateral subcortical ischemic stroke based on magnetic resonance imaging (MRI) findings, ③ patients with chronic disease stage (>3 months and <12 months), ④ patients with mild or moderate impairment of upper limb function (Upper Extremity Fugl-Meyer Assessment [UEFM] score of 40–60), or ⑤ patients had normal cognitive function, as confirmed by a Montreal Cognitive Assessment (MoCA) score >25. The exclusion criteria of the patients were as follows: ① patients with a history of severe heart, liver, lung, kidney, and hematopoietic primary disease or epilepsy; ② patients with unstable cardiovascular and cerebrovascular diseases; ③ patients with visual analog scale score of the shoulder >4; ④ patients with severe upper limb dystonia and joint contracture deformity; ⑤ patients with metallic implants in the head, pacemakers, history of seizure or epilepsy, significant psychiatric conditions and other conditions which is not suitable for tDCS intervention as described by Antal et al. (12); ⑥ patients inability to commit to the full study protocol as determined during the initial screening interview, or failure to complete a preliminary compliance check session.

### 2.2 Experimental design

**Figure 2:**
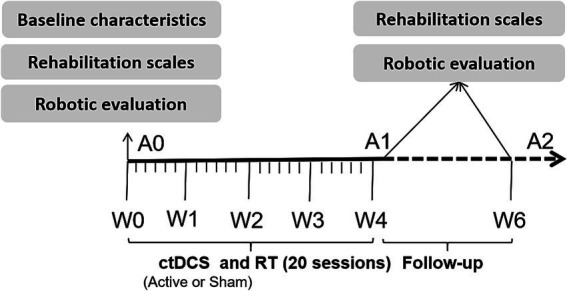
Experimental protocol sequence of this study.

This was a randomized controlled trial comparing the efficacy of combined RT and active ctDCS versus RT plus sham ctDCS for improving upper limb function in stroke patients. Patients were randomly assigned (according to the random number table) to receive a total of 20 sessions during 4 weeks (1 session per day, 5 days/week) of active or sham ctDCS during upper limb RT. After each session, a questionnaire was administered to determine the patients’ symptoms (including prickliness, itching, burning, pain, headache, fatigue, inattention, and anxiety) (12) and degree (score ranging from 1 to 5) during the tDCS intervention. Additionally, the Borg Fatigue Assessment scale (6–20) was used to evaluate the patients’ degree of fatigue after RT. Patients were blinded to their group allocation (active or sham ctDCS). The therapists administering the tDCS could not be blinded due to the operational requirements of the device. However, the assessors who conducted all clinical and robotic evaluations were blinded to the group allocation throughout the study. Figure 2 shows the experimental protocol sequence.

#### 2.2.1 Cathodal transcranial direct current stimulation protocol

Cathodal ctDCS was delivered using a battery-driven constant DC stimulator (neuroConn, Germany) with a pair of 35 cm^2^ (5 cm × 7 cm) electrodes inserted into saline-soaked sponges. Active stimulation was delivered at 1.75 mA (current density, 0.5 A/m^2^) for 20 min (13). In sham tDCS, the current was delivered only for the first 43 s (8 s ramp-up, 30 s of DC stimulation, and 8 s ramp-down) to make the participants feel a tingling sensation at the beginning of the stimulation (14). ctDCS was performed with the cathode placing over the M1 of the unlesioned hemisphere and the anode placing over the ipsilesional supraorbital region. The location of M1 was set as C3 (left hemisphere) or C4 (right hemisphere) according to the international 10–20 electroencephalogram (EEG) electrode placement system. The above protocol was consistent with our previous study (8).

#### 2.2.2 Robotic therapy protocol

**Figure 3:**
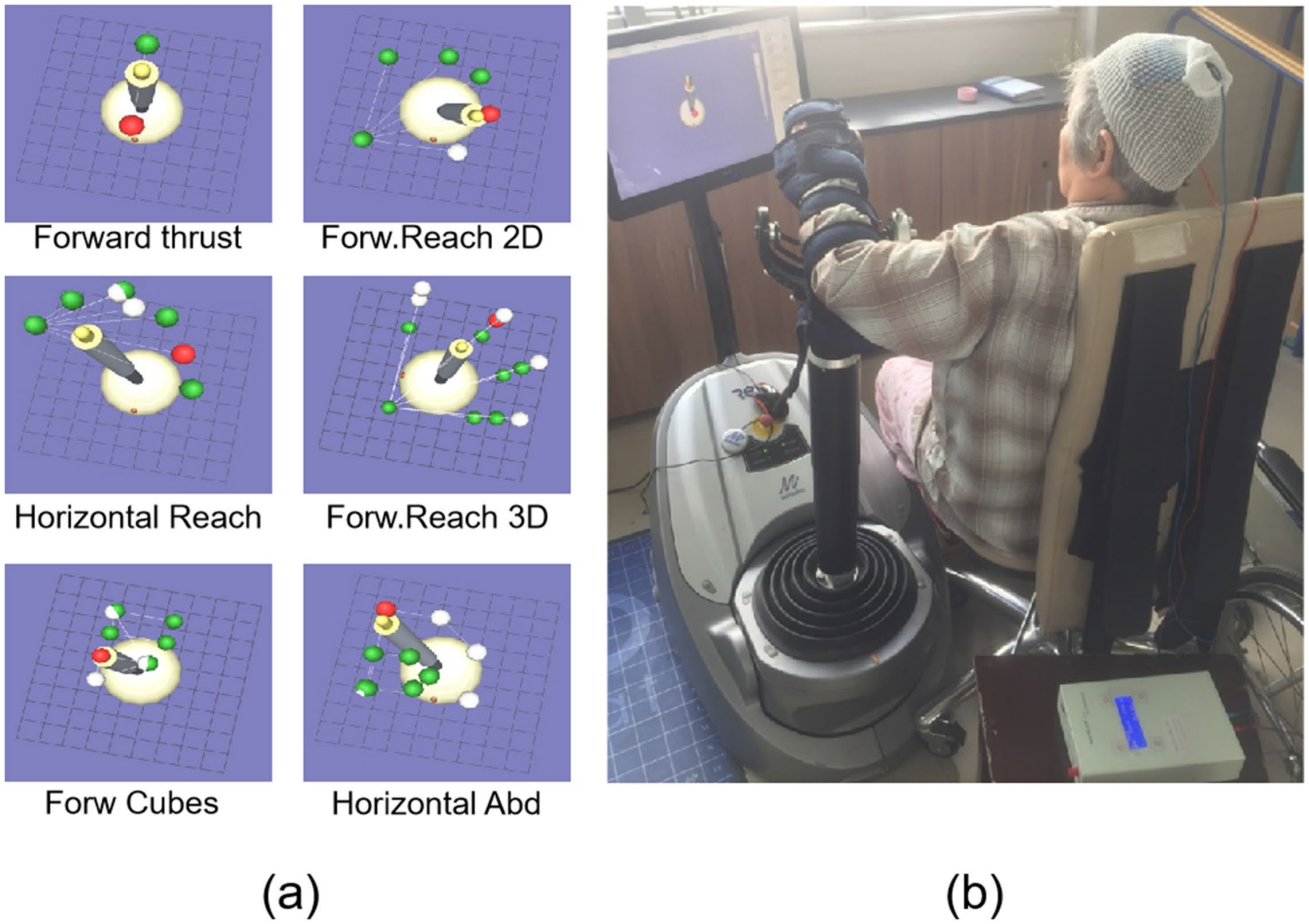
Trajectory of robotic motion **(a)** and combined treatment scene of cathodal transcranial direct current stimulation and robot training **(b)**.

The RT protocol was conducted using an upper limb RT system (ReoGo, United States). The efficacy of this system in improving the UE function has been established in two RCTs (15, 16). The device consists of a telescopic arm with three-axis force-sensing technology, enabling it to support and guide the patient’s upper limb through movements in three-dimensional space. We selected six distinct motion trajectories designed to train functional movement patterns involving shoulder flexion/extension, abduction/adduction, and combined shoulder-elbow coordination (Figure 3). The device offers five training modes, which are selected by the therapist based on the patient’s residual motor capability: ① Guide: The robot moves the patient’s limb through the entire trajectory without any voluntary effort from the patient. ② Initiate: The patient must initiate movement by generating a minimal force threshold at the starting position; upon triggering, the robot completes the remainder of the movement. ③ Step-Initiate: The patient must voluntarily initiate movement and subsequently hit force thresholds at multiple points along the trajectory to complete the movement. ④ Follow-assist: The patient must provide continuous active effort to complete the movement. The robot provides a constant, adjustable level of assistance to compensate for weakness. ⑤ Free: The patient moves actively against adjustable resistance provided by the robot to strengthen muscles. The device has three force levels (low, medium, and high) representing the magnitude of resistance. The specific parameters for each trajectory and assistance mode are detailed in Table 1. The assistance level was set to provide minimal necessary support to complete the movement smoothly, while the resistance level in active mode was set to a level that allowed for 8–12 repetitions per set without compensatory movements. The range of motion was standardized to 90% of the patient’s available passive range to prevent discomfort or injury. Each RT session lasted 30 min.

**Table 1:** Description of robotic therapy training trajectories and parameters.

Trajectory No.	Movement description	Targeted joint movement and ROM	Force levels	Assistance mode
1	Forward thrust	Shoulder horizontal abduction (0–10°), adduction (0–70°), with elbow flexion and extension (0–120°)	Low, medium, high	Guide, Initiate, Step-Initiate, Follow-assist, Free
2	Forward Reach-2D	Shoulder horizontal adduction (0–90°), with elbow flexion and extension (0–130°)	Low, medium, high	Guide, Initiate, Step-Initiate, Follow-assist, Free
3	Horizontal Reach	Shoulder horizontal abduction (0–30°) and adduction (0–70°), with elbow flexion and extension (0–150°)	Low, medium, high	Guide, Initiate, Step-Initiate, Follow-assist, Free
4	Forward Reach-3D	Shoulder flexion (0–130°); shoulder horizontal adduction (0–90°), with elbow flexion and extension (0–150°)	Low, medium, high	Guide, Initiate, Step-Initiate, Follow-assist
5	Forward Cubes	Shoulder horizontal adduction (0–90°), with elbow flexion and extension (0–150°).	Low, medium, high	Guide, Initiate, Step-Initiate, Follow-assist, Free
6	Horizontal Rotation	Shoulder flexion (0–120°), horizontal adduction (0–90°), with elbow flexion and extension (0–150°).	Low, medium, high	Guide, Initiate, Step-Initiate, Follow-assist

ROM, range of motion; In 3D mode (Trajectory No.3 and No.6), the free mode cannot be used due to the characteristics of the device’s control mechanism.

### 2.3 Clinical outcome measurements

Clinical outcome measurements, including upper limb rehabilitation scales and robotic evaluation, were conducted before, after, and at follow-up (2 weeks after the completion of training).

#### 2.3.1 Rehabilitation scales

The UEFM scale was used to evaluate upper limb motor recovery (17). The Box and Block Test (BBT) was used to quantify gross manual dexterity, assessed by the number of blocks transported unilaterally within one minute (18), offering a rapid, objective metric of gross motor speed and coordination. The Jebsen-Taylor Hand Function Test (JTHF) was employed to evaluate a broader spectrum of functional abilities by timing the performance of simulated activities of daily living (19), assessing integrated fine and gross motor skills required for functional tasks. Higher UEFM scores and BBT indicate better upper limb motor function, and lower JHFT indicate better upper limb motor function. All the above measurements were conducted by a rehabilitation therapist blinded to the grouping and intervention.

#### 2.3.2 Robotic evaluation

The horizontal reach trajectory from the same robotic system was used to quantitatively measure three key upper limb parameters: movement velocity (Speed), range of motion (Scale), and integrated functional capability (Score) in poststroke patients. The first two parameters Speed and Score were assessed under the Follow-assist mode with low force level. This mode was selected based on two considerations: first, it ensured all patients could complete the assessment; second, it effectively differentiated functional abilities among patients. The parameter “Speed” characterizes the movement velocity of the upper limb, while the parameter “Scale” represents the active range of motion of the patient’s joints. Both parameters were assessed at the patient’s maximal tolerance level to capture peak performance. Additionally, we developed a composite scoring system to quantitatively assess patients’ motor performance during robotic-assisted evaluation. The scoring method was constructed as follows: Five assistance modes were assigned baseline scores of 10, 20, 30, 40, and 50 points, corresponding to increasing levels of task difficulty. Additionally, the range of motion (ROM) achieved during each task was incorporated into the score: for every 10% increase in ROM beyond the baseline, 2 points were added, while a 10% decrease resulted in a deduction of 2 points. Furthermore, three distinct force levels (low, medium, high) were available within each training mode, which were assigned scores of 0, 2, and 4 points, respectively. The final performance score for each patient was calculated by summing three components: the score of the highest difficult training mode they could successfully complete, along with the corresponding ROM adjustment points and force level points achieved during the assessment. While this composite scoring system is novel, it was designed to integrate key motor performance dimensions (task difficulty, range of motion, and force control) into a single quantitative metric. The construct validity of this score is supported by its significant correlation with established clinical scales of the Fugl-Meyer Assessment (with baseline data) (Pearson correlation coefficient *r* = 0.761, *p* < 0.001) in our cohort.

### 2.4 Statistical analyses

This study used the per-protocol analysis method for the analysis, with the aim of evaluating the efficacy of the combined use of ctDCS and RT under ideal conditions. Statistical analyses were performed using SPSS version 21.0, with statistical significance set at *p* < 0.05. An independent *t*-test was used to compare the differences in age, poststroke time, and baseline assessment scale scores between the active and sham groups. The chi-squared test was used to compare differences in sex, paretic side, and lesion sites. All data used for the independent *t*-test were normally distributed according to the Kolmogorov–Smirnov test.

We used repeated measures analysis of variance (ANOVA) with time (three levels: pre, post, and follow-up) as the within-subject factor and group (two levels: active and sham) as the between-subject factor to evaluate the difference in the rehabilitation outcome measurement scale and robotic evaluation scale over the treatment and follow-up. Before the ANOVAs, Mauchly’s test of sphericity was used to test covariance matrix sphericity. If the spherical assumption was not satisfied, the Greenhouse–Geisser method was used to adjust the degree of freedom to reduce the probability of a type I error. A Bonferroni-corrected *post hoc* comparison was conducted after repeated ANOVA.

## 3 Results

### 3.1 Demographic information

Demographic information of the patients is shown in Table 2. There were no significant differences in age, poststroke time, or sex between the two groups (*p* > 0.05). The questionnaire regarding symptoms during tDCS showed no differences in the side effects of active and sham stimulations. The two groups also showed no significant differences in fatigue after RT according to the Borg fatigue scale.

**Table 2:** Participant characteristics in this study.

Variables	Active group (*n* = 16)	Sham group (*n* = 15)	χ^2^/*T*	*p*-value
Sex, male/female (%)	13/3 (81.3%)	8/7 (53.3%)	2.761	0.097
Age, year (mean ± SD)	55.63 ± 9.30	62.00 ± 8.68	−1.970	0.058
Paretic side, right/left (%)	7/9 (56.3%)	8/7 (46.7%)	0.285	0.594
Lesion site, BG/BS (%)	13/3 (81.3%)	9/6 (60.0%)	1.697	0.193
Poststroke time (/months)	8.75 ± 2.86	8.80 ± 2.73	−0.050	0.961
UEFM (mean ± SD)	54.63 ± 7.50	49.67 ± 6.62	1.946	0.061
BBT (mean ± SD)	7.50 ± 2.80	6.80 ± 2.46	0.737	0.467
JHFT (mean ± SD)	73.65 ± 18.78	77.07 ± 18.63	−0.511	0.613

BG, basal ganglia; BS, brain stem; UEFM, Upper Extremity Fugl-Meyer; BBT, Box and Block Test; JHFT, Jebsen–Taylor Hand Function Test; χ^2^, chi-squared; *T*, independent sample *t*-test.

### 3.2 Rehabilitation scales

The results of the repeated measures ANOVA with time as the within-subject factor and group as the between-subject factor are presented in Table 3. It revealed significant within-subject improvements across time for all three rehabilitation scales: the UEFM, the BBT, and the JHFT (all *p* < 0.001). For between-subject effects, a significant difference was found only in the UEFM scores between the groups (*p* = 0.023). Furthermore, a significant time-by-group interaction was observed for the BBT scores (*p* = 0.033), indicating that the rate of improvement over time differed between the groups. The results of the post-hoc comparison are shown in Table 4. For UEFM, the between-group results showed that the UEFM scores were significantly different at post-treatment (*p* = 0.013) and follow-up (*p* = 0.015), and within-group results showed that the active group had a significant difference both post-treatment (*p* = 0.029) and at follow-up (*p* = 0.026). For the BBT, between-group results showed that BBT scores were significantly different at post-treatment (*p* = 0.038) and follow-up (*p* = 0.017), and within-group results showed significant pairwise differences at each of the three time point in both the active and sham groups.

**Table 3:** Repeated measures analysis of variance results of rehabilitation scales.

Rehabilitation Scale	Group	Pre	Post	Follow-up	Time			Group			Time × group		
					*F*	sig	η2	*F*	sig	η2	*F*	sig	η2
UEFM	Active	54.63 ± 7.50	56.88 ± 5.16	55.94 ± 5.62	12.041	**0.001**	0.293	5.753	**0.023**	0.166	0.068	0.844	0.002
	Sham	49.67 ± 6.62	52.27 ± 4.42	51.07 ± 4.85									
BBT	Active	7.19 ± 2.66	12.69 ± 3.65	11.81 ± 3.67	122.963	**<0.001**	0.809	2.070	0.161	0.067	3.630	**0.033**	0.111
	Sham	6.73 ± 2.19	10.87 ± 2.92	9.73 ± 2.46									
JHFT	Active	78.01 ± 15.33	63.65 ± 10.50	66.00 ± 11.93	28.354	**<0.001**	0.494	1.378	0.250	0.045	3.481	0.068	0.107
	Sham	78.73 ± 17.18	72.17 ± 11.53	72.25 ± 11.39									

Bold represents that *p* < 0.05.

**Table 4:** *P* value of pos-hoc comparison results of rehabilitation scales after repeated measures analysis of variance.

Scale		Comparison					
		Time			Group		
		Pre vs. Post	Post vs. Follow-up	Pre vs. Follow-up	Pre	Post	Follow-up
UEFM	Active	**0.029**	**0.026**	0.142	0.061	**0.013**	**0.015**
	Sham	0.058	0.093	0.163			
BBT	Active	**<0.001**	**0.024**	**<0.001**	0.609	**0.038**	**0.017**
	Sham	**<0.001**	**0.040**	**<0.001**			
JHFT	Active	**<0.001**	**0.002**	**<0.001**	0.902	0.060	0.147
	Sham	**0.033**	0.087	**0.043**			

Bold represents that *p* < 0.05.

The observed effect size of primary outcome measure was 0.65, calculated using Cohen’s d. This was derived by dividing the mean difference in post-intervention UEFM scores between the active group (M = 56.88, SD = 5.16, *n* = 16) and the sham group (M = 52.27, SD = 4.42, *n* = 15) by the pooled standard deviation of the groups’ baseline scores (SD_pooled_baseline = 7.09). This approach, which uses baseline variability for standardization, is recommended to minimize potential bias introduced by the intervention itself and provides a conservative estimate of the effect.

### 3.3 Robotic evaluation

The results of the repeated measures ANOVA with time as the within-subject factor and group as the between-subject factor are presented in Table 5. The three robotic evaluation scales (scale, speed, and score) showed significant within-subject differences (*p* < 0.001). Only speed scale presented a significant interaction between time and group (*p* = 0.023). The results of post-hoc comparison are shown in Table 6. Post-hoc pairwise comparisons show significant pairwise differences at each of the three time point in both the active and sham groups, and only the speed was significantly different between groups at post-treatment (*p* = 0.035) and follow-up (*p* = 0.043).

**Table 5:** Repeated measures analysis of variance results of robotic evaluation.

Robotic Scale	Group	Pre	Post	Follow-up	Time			Group			Time × group		
					*F*	sig	η2	*F*	sig	η2	*F*	sig	η2
Scale	Active	88.13 ± 7.93	117.50 ± 10.80	113.13 ± 9.81	239.989	**<0.001**	0.892	1.795	0.191	0.058	1.637	0.211	0.053
	Sham	87.00 ± 6.49	112.33 ± 10.67	107.67 ± 8.63									
Speed	Active	49.06 ± 7.79	58.13 ± 6.29	55.31 ± 4.64	33.054	**<0.001**	0.533	0.909	0.348	0.03	4.905	**0.023**	0.145
	Sham	50.33 ± 5.82	54.67 ± 4.42	52.33 ± 4.17									
Score	Active	34.00 ± 6.93	42.13 ± 4.47	40.13 ± 3.61	71.833	**<0.001**	0.712	1.677	0.206	0.055	0.962	0.356	0.032
	Sham	32.53 ± 6.82	39.20 ± 5.44	37.07 ± 6.32									

Bold represents that *p* < 0.05.

**Table 6:** *P* value of pos-hoc comparison results of robotic evaluation after repeated measures analysis of variance.

Scale		Comparison					
		Time			Group		
		Pre vs. Post	Post vs. Follow-up	Pre vs. Follow-up	Pre	Post	Follow-up
Scale	Active	**<0.001**	**0.001**	**<0.001**	0.670	0.191	0.112
	Sham	**<0.001**	**0.001**	**<0.001**			
Speed	Active	**<0.001**	**<0.001**	**<0.001**	0.613	**0.035**	**0.043**
	Sham	**0.001**	**0.021**	0.514			
Score	Active	**<0.001**	**0.001**	**<0.001**	0.558	0.112	0.106
	Sham	**<0.001**	**<0.001**	**0.001**			

Bold represents that *p* < 0.05.

## 4 Discussion

This study investigated the effectiveness of a combined ctDCS and RT intervention for improving upper limb function in patients with chronic ischemic stroke. The results of the rehabilitation scales showed similar improvement trends (significant improvement post-treatment and some reduction in the follow-up) in both the active and sham groups, but the rehabilitation effect in the active group was better than that in the sham group. The results of the robotic evaluation showed the advantage of the active group in increasing movement speed.

Previous study reports that after unilateral hemispheric stroke, there is a pathological decrease in excitation in the ipsilesional M1 and an increase in the contralesional M1. In addition, the overactive contralesional M1 exerts exaggerated interhemispheric inhibition on the ipsilesional M1 and further aggravates the residual motor function of the paretic arm (20). Based on this, using anodal tDCS to increase the excitability of the ipsilesional M1, ctDCS to decrease the excitability of the contralesional M1 or use both at the same time can promote rehabilitation process poststroke (8).

Anodal polarity placed on the ipsilesional M1 is one of the most common tDCS protocols for promoting upper limb function poststroke. Several studies have demonstrated its effect on M1 activation, as assessed by functional MRI (21) and EEG (8, 22). However, its efficacy in promoting upper limb motor function remains unclear, with clinical trials reporting heterogeneous outcomes. Studies such as (38) and Triccas et al. (23) reported significant functional improvements, particularly in subacute stroke patients and those with subcortical lesions—populations with relatively preserved corticospinal infrastructure. This suggests that atDCS may preferentially facilitate recovery in circuits with residual structural and functional integrity. Some study reported that the effect of combined use of atDCS and RT was limited especially in chronic stroke (23). This inconsistency may stem from injury location and severity, and the timing of the intervention relative to the stroke onset.

Bilateral tDCS aims to restore interhemispheric balance by simultaneously upregulating excitability in the ipsilesional M1 and downregulating the contralesional M1. This dual modulation may normalize pathological interhemispheric inhibition (IHI), thereby creating a permissive environment for use-dependent plasticity during motor training. However, its clinical effects to upper limb recovery is inconsistent. Our previous study found no significant improvement of upper limb function in poststroke subjects after bilateral tDCA (8, 39) reported that the effect of bilateral tDCS combined with RT depending on the duration and type of stroke, and patients with subcortical and chronic lesions exhibited greater improvement.

Some studies focused on cathodal tDCS. By applying cathodal stimulation to the contralesional hemisphere, ctDCS may help normalize the imbalance between the two hemispheres and promote adaptive plasticity in the damaged motor cortex. This, in turn, may facilitate motor recovery and functional reorganization within the ipsilesional hemisphere, leading to improvements in the upper limb function (8, 22). Additionally, ctDCS has a more pronounced effect on cortical excitability than atDCS, with studies demonstrating greater reductions in motor-evoked potentials and increased motor thresholds following cathodal stimulation (24). This suggests that ctDCS may induce more robust changes in the cortical physiology, potentially making it more effective in modulating neural activity and promoting motor rehabilitation. Some studies have compared the rehabilitation effect of ctDCS over the contralesional M1 with that of anodal tDCS over the ipsilesional M1, indicating no significant difference in the improvement effect between the two groups after 10 days of stimulation but a higher improvement in ctDCS 6 months after treatment (25). This study further confirmed the effect of ctDCS on the upper limb function in patients with stroke.

Incorporating repetitive rehabilitation training, motor relearning, and other training techniques while stimulating the M1 area of the cerebral cortex with tDCS can significantly improve the motor function of the upper limbs, and this improvement is sustained (9, 25). A previous study showed that, compared with occupational therapy alone, the combination of ctDCS over the contralesional M1 and occupational therapy could improve upper limb motor function more effectively, and the improvement effect could last for at least 1 week. Meanwhile, the functional activity of the contralateral motor cortex decreased (26).

Intensified task-specific training, which is repetitive, challenging, and functionally meaningful for patients, is highly effective for upper limb recovery after stroke (3). RT can control the task learning phase more easily than traditional therapeutic techniques because robots allow patients to perform guided movements on predefined pathways and avoid possible uncontrolled movements (5). Clinical studies using different designs and methods have verified the rehabilitation effects of robotic devices on the upper and lower limbs (27).

Researchers have conducted studies on the combination of ctDCS and RT to recover upper limb function in patients with stroke. However, there is no consensus on the effectiveness of this combined treatment. Hesse et al. (28) found that after combined treatment with ctDCS and RT, patients with subacute stroke with subcortical lesions achieved better rehabilitation effects than did those with cortical lesions. This is consistent with the results obtained in our study on patients with chronic subcortical infarction stroke. Ochi et al. (29) reported that ctDCS was more suitable than atDCS for patients with right hemispheric stroke. This is consistent with the results obtained in our study on patients with chronic subcortical infarction stroke. The present study demonstrated the advantages of the combined treatment in improving the movement speed of some upper limb motor tasks. However, some studies have found no significant difference in improving upper limb function between the combined treatment of ctDCS and RT and RT alone (29, 30). Possible factors that may lead to variable clinical results include the heterogeneity of the stroke population (i.e., chronic or acute, ischemic or hemorrhagic, cortical or subcortical), different stimulation protocols, RT devices, and outcome measures (4, 5).

Our study demonstrated that the combination of ctDCS and robotic therapy (RT) led to clinically meaningful improvements in upper limb function, surpassing the effects of RT alone. We interpret this result as evidence of a synergistic effect rather than a mere additive effect. ctDCS is believed to modulate cortical excitability and create a state of heightened plasticity in the targeted motor network (31). This “priming” effect means that the neural circuits involved in hand and arm movements are more receptive to the subsequent motor learning induced by RT (32, 33). Robotic therapy provides high-intensity, repetitive, and task-oriented training. This drives use-dependent plasticity, reinforcing the neural pathways that control movement. When applied during ctDCS, this reinforcement is potentiated. The brain is not just practicing. It is learning more efficiently and effectively due to the primed state (34). Additionally, in stroke recovery, an imbalance between the two hemispheres is common. By applying cathodal stimulation to the unaffected, ctDCS may help rebalance the inhibitory interhemispheric interactions (35). This rebalancing could allow the affected hemisphere to generate motor commands with less inhibition, thereby enabling the patient to engage more fully and successfully with the RT, leading to greater functional gains.

Another important consideration is the role of spasticity in motor recovery. As spasticity is a common complication arising from disrupted interhemispheric inhibition post-stroke, our chosen intervention of cathodal tDCS over the contralesional hemisphere may have indirectly addressed spasticity. By inhibiting the hyperexcitable contralesional motor cortex, cathodal tDCS could potentially reduce excessive spinal reflex activity and contribute to a decrease in spasticity (36). This reduction could, in turn, facilitate improved voluntary movement and enhance the benefits of robotic training, representing a possible mechanism underlying the functional gains observed in our study.

However, spasticity was not formally assessed in this study. While patients with severe spasticity that would prevent engagement with the robotic device were not enrolled, the presence of varying degrees of spasticity in our cohort was not quantified using scales such as the Modified Ashworth Scale. This constitutes a limitation, as spasticity may have influenced movement quality and functional outcomes independently of the intervention’s effects on voluntary motor control. Future studies should incorporate standardized spasticity assessments to disentangle its effects from those on active motor function. Additionally, the relatively small sample size limits the statistical power and generalizability of our findings, and single blinding procedure may introduce potential for delivery bias.

Based on the limitations of the study, a follow-up study with an expanded sample size will be conducted to confirm the clinical efficacy of the combined ctDCS and Robotic Therapy (RT) intervention. Additionally, future work will incorporate standardized spasticity assessments, such as the Modified Ashworth Scale, to disentangle the intervention’s effects on voluntary motor control from its potential indirect impact on reducing spasticity. This distinction is crucial, as spasticity may independently influence movement quality and functional outcomes. Finally, elucidating the neuroplastic mechanisms underlying functional improvements is a key objective. This will be achieved by adding more assessment timepoints and employing more frequent neurophysiological (e.g., TMS, EEG) or kinematic measures. The goal is to understand how ctDCS primes the brain for learning and interacts with RT-driven, use-dependent plasticity.

## 5 Conclusion

The findings of this study suggest that combining ctDCS with RT may enhance the efficiency of specific upper limb motor tasks in patients with chronic subcortical ischemic stroke, compared to RT alone.

## Data Availability

The datasets presented in this article are not readily available because the data is restricted by the Tianjin Union Medical Centre, in order to protect patients’ privacy. Data is available from corresponding author of the paper for researchers who meet the criteria for access to the confidential data. Requests to access the datasets should be directed to chfwang@tju.edu.cn.
